# Heart Rate Variability and Cerebral Autoregulation in Patients with Traumatic Brain Injury with Paroxysmal Sympathetic Hyperactivity Syndrome

**DOI:** 10.1007/s12028-024-02149-1

**Published:** 2024-10-29

**Authors:** Małgorzata Burzyńska, Jowita Woźniak, Piotr Urbański, Jarosław Kędziora, Rafał Załuski, Waldemar Goździk, Agnieszka Uryga

**Affiliations:** 1 Clinical Department of Anesthesiology and Intensive Care, Faculty of Medicine, Wroclaw Medical University, Wrocław, Poland; 2 Clinical Department of Neurosurgery, Faculty of Medicine, Wroclaw Medical University, Wrocław, Poland; 3 Department of Biomedical Engineering, Faculty of Fundamental Problems of Technology, Wroclaw University of Science and Technology, Wybrzeze Wyspianskiego 27, 50-370 Wrocław, Poland; 4 Department of Neurosurgery, Wroclaw University Hospital, Wroclaw, Poland

**Keywords:** Nervous system, Heart rate, Baroreflex, Brain injuries

## Abstract

**Background:**

Severe traumatic brain injury (TBI) can lead to transient changes in autonomic nervous system (ANS) functioning and development of paroxysmal sympathetic hyperactivity (PSH) syndrome. Clinical manifestation of ANS disorders may be obscured by therapeutic interventions in TBI. This study aims to analyze ANS metrics and cerebral autoregulation in patients with PSH syndrome to determine their significance in early prognostication.

**Methods:**

This single-center retrospective study investigated the relationship between changes in ANS metrics, cerebral autoregulation, and PSH syndrome. Arterial blood pressure and intracranial pressure signals were monitored for 5 days post TBI. ANS metrics included time and frequency domain heart rate variability (HRV) metrics. Cerebral autoregulation was assessed using the pressure reactivity index.

**Results:**

Sixty-six patients with severe TBI (median age 33 [interquartile range 26–50] years) were analyzed, and PSH was confirmed in nine cases. Impairment of cerebral autoregulation was observed in 67% of patients with PSH and 72% without the syndrome. Patients with PSH had higher HRV in the low-frequency range (LF; 253 ± 178 vs. 176 ± 227 ms^2^; *p* = 0.035) and lower heart rates (HRs; 70 ± 7 vs. 78 ± 19 bpm; *p* = 0.027) compared to those without PSH. A receiver operating characteristic curve analysis indicated that HR (area under the curve (AUC) = 0.73, *p* = 0.006) and HRV in the LF (AUC = 0.70, *p* = 0.009) are moderate predictors of PSH. In the multiple logistic regression model for PSH, diffuse axonal trauma (odds ratio (OR) = 10.82, 95% confidence interval (CI) = 1.70–68.98, *p* = 0.012) and HR (OR = 0.91, 95%  CI 0.84–0.98, *p* = 0.021) were significant factors.

**Conclusions:**

Elevated HRV in the LF and decreased HR may serve as early predictors of PSH syndrome development, particularly in patients with diffuse axonal trauma. Further research is needed to investigate the utility of the cerebral autoregulation–ANS relationship in PSH prognostication.

**Supplementary Information:**

The online version contains supplementary material available at 10.1007/s12028-024-02149-1.

## Introduction

Traumatic brain injury (TBI) is a major medical and socioeconomic challenge worldwide. The annual incidence of TBI is estimated to be around 69 million cases worldwide. In the United States alone, there are about 2.53 million cases each year, whereas in the European Union, there are 1.5 million hospitalizations and 57,000 deaths per year [1]. Mortality in the TBI population is significantly higher than that in the general population, constituting approximately 37% of all injury-related deaths [2]. Furthermore, the life expectancy of trauma survivors is reduced to 40–85% of that in the general population, and this reduction is linked to a significant decline in quality of life [3]. TBI can result in enduring limitations in daily functioning due to impaired motor function, behavioral and cognitive changes, and increased incidence of mental health issues, such as depression, amnesia, anxiety, and posttraumatic stress disorder [4–6].

The outcome of patients with TBI is influenced not only by the severity of brain damage but also by the extent of systemic disorders and the function of peripheral organs. Recent studies have shown that autonomic nervous system (ANS) disorders may significantly affect the overall outcome [7–9]. The ANS modulates the systemic response to the central inflammatory process caused by TBI through the hypothalamic–pituitary axis and the immune system [10–12]. Its disruption may result in impairment of extracranial organ function, cardiovascular dysregulation, and metabolic and immune dysfunction, ultimately leading to an increased systemic inflammatory response and multiorgan failure [13]. Assessment of ANS function is performed using heart rate variability (HRV), which describes the variation in time intervals between heartbeats [14], and baroreflex sensitivity (BRS), defined as the changes in interbeat intervals per unit change in systolic blood pressure [15].

Impaired cardiovascular regulation and a shifted balance between sympathetic and parasympathetic outflows have been reported in previous studies of TBI and acute brain injury populations [16–18]. Increased sympathetic nervous system activity is reported in approximately 8–33% of patients with TBI, manifesting as paroxysmal sympathetic hyperactivity (PSH) [19]. PSH is characterized by recurrent episodes of elevated heart rate (HR), blood pressure, respiratory rate, and temperature; sweating; and motor (posturing) activity [20]. It has been observed across all stages of brain injury, from early critical care to the rehabilitation phase. The diagnosis of PSH relies on the 2014 standardized Clinical PSH Assessment Measure (PSH-AM) scoring system, which also serves as a valuable tool for assessing the severity of PSH and monitoring the effectiveness of PSH treatment [21, 22]. However, it is essential to acknowledge the nonspecific nature of PSH-AM, posing a potential challenge in the intensive care unit (ICU) setting. Therefore, timely and accurate diagnosis and early treatment may be crucial in improving outcomes for patients with TBI.

Another important and still little-known issue is the relationship between ANS and cerebral autoregulation. Cerebral autoregulation is a key physiological process that maintains steady cerebral blood flow (CBF) despite changes in mean arterial pressure [23]. The unique structure of the cerebral vasculature, the need to control cerebral blood volume and intracranial pressure (ICP), and the necessity to ensure optimal delivery of oxygen and nutrients to the brain make CBF regulation a complex process that depends on both systemic and internal factors, including the ANS [24]. The diverse distribution of adrenergic receptors in the cerebral vasculature makes the response to sympathetic stimulation highly dependent on the type of receptor, its density in the vessel, the concentration of neurotransmitters released, and the presence of other vasoactive compounds [25]. The specificity of sympathetic regulation, which varies by region within the cerebral unit, also applies to parasympathetic innervation. The dense parasympathetic innervation of cholinergic nerve fibers in the cerebral vasculature may influence the magnitude of CBF and cerebral autoregulation [26–28]. Hoiland and Ainslie [29] suggest that a balance between sympathetic and parasympathetic reflexes optimizes CBF in response to both rising and falling blood pressure. However, it has been shown that cerebral autoregulatory vasodilation can still occur in patients with autonomic failure [30] and remain intact in patients with severely compromised autonomic function [31]. Understanding the relationship between ANS and cerebral autoregulation is the subject of ongoing debate and may be an important step in improving TBI care [32–34].

In this study, we investigated the relationship between ANS activity and incidence of PSH, aiming to determine the utility of autonomic metrics as significant risk factors. Moreover, we analyzed cerebral autoregulation in patients with PSH and described the relationship between ANS and cerebral autoregulation in the early period after TBI. We hypothesized that impairment of both ANS and cerebral autoregulation in the early period after TBI in patients undergoing standard intensive care treatment may influence poorer prognosis and morbidity.

## Methods

### Data Collection and Patient Management

**Fig. 1:**
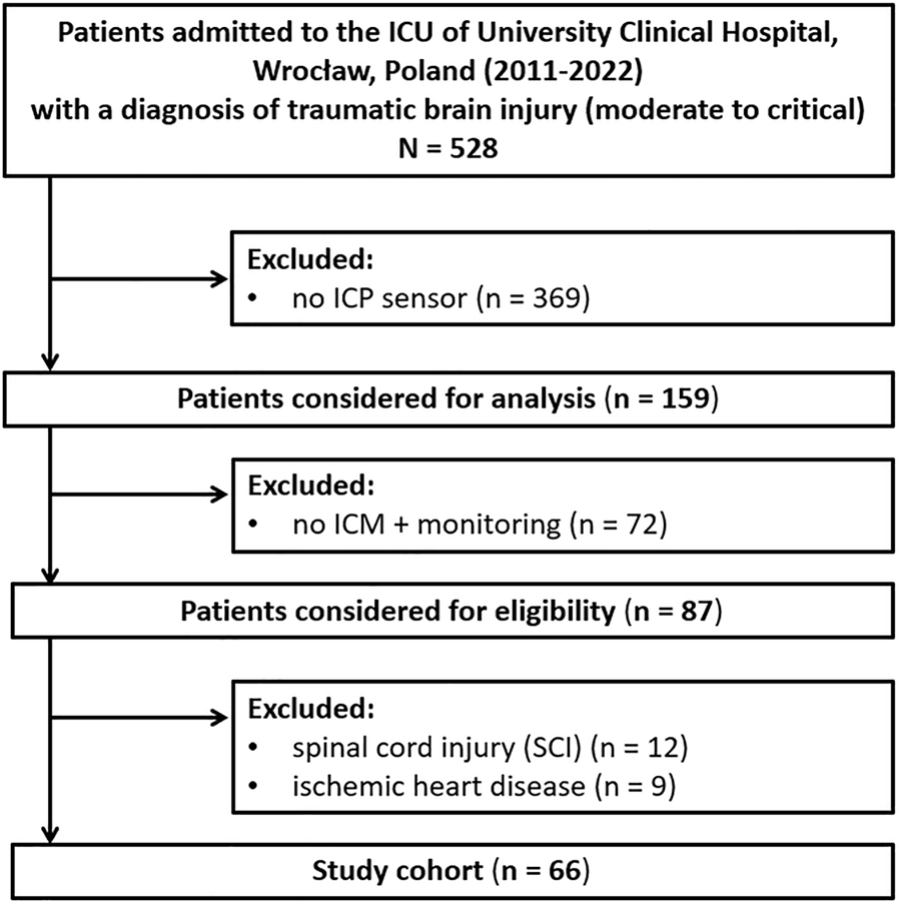
Flowchart. ICM+, Intensive Care Monitor software; ICP, intracranial pressure; ICU, intensive care unit

This study retrospectively analyzed the data of patients with moderate to severe TBI admitted to the ICU of the University Clinical Hospital in Wroclaw between 2011 and 2022. The study was approved by the Bioethics Committee of the Wroclaw Medical University (KB-133/2023; waived the need for informed consent) and conducted according to the Strengthening the Reporting of Observational Studies in Epidemiology (STROBE) statement for observational cohort studies (Supplementary materials). The following inclusion criteria were used: (1) age of 18 years or older; (2) moderate or severe TBI, assessed by the Glasgow Coma Scale (GCS) as an isolated injury or predominate multiorgan injury [35]; (3) implementation of the ICP sensor; and (4) hemodynamically stable patient when monitoring started. Exclusion criteria were as follows: (1) previous or concurrent diagnoses that could explain symptoms of autonomic dysfunction (e.g., long-term diabetes, ischemic heart disease), (2) concurrent traumatic spinal cord injury [36], and (3) lack of good-quality, high-resolution frequency signal recordings initiated within 24 h after ICU admission. The flowchart is shown in Fig. 1.

After the initial trauma assessment and stabilization of the patient in the emergency department based on Advanced Trauma Life Support principles of airway, respiration, and circulation [37], patients were admitted directly to the ICU or postoperatively after urgent surgery or ICP sensor placement. All patients underwent multimodal monitoring, including arterial blood pressure (ABP), ICP, and cerebral perfusion pressure (CPP) signals. Patients were treated according to the head injury treatment guidelines in effect at the time of admission [38, 39] and the polytrauma guidelines [37]. During the entire follow-up period, which included the first 5 days of the patient’s stay in the ICU, all patients were sedated and intubated and received mechanical ventilation. An ICP/CPP management algorithm was applied to all patients, aiming to achieve ICP below 20 to 22 mm Hg and CPP above 60 mm Hg using therapeutic interventions (e.g., osmotic agents, moderate hyperventilation, vasopressors, intravenous fluid temperature control, and decompressive craniotomy if necessary).

The initial neurological status of each patient was determined using the GCS on admission to the hospital. We defined the severity of head injury using the Mayo Clinic classification for TBI severity [40], in which moderate to severe TBI was diagnosed if one or more of the following criteria applied: posttraumatic amnesia lasting more than 24 h; loss of consciousness lasting more than 30 min; GCS score less than 13 in the first 24 h (excluding, e.g., intoxication, sedation, systemic shock); death due to TBI; and more than one of the following: subarachnoid hemorrhage, intracranial hematoma, brain contusion or hemorrhage, dura mater puncture, and brainstem injury. The Injury Severity Scale was used to quantify the severity of the injury according to the Berlin definition. It is calculated as a composite score based on an Abbreviated Injury Scale score ≥ 3 for two or more different body regions in conjunction with one or more additional variables from the five physiological parameters of age, consciousness, hypotension, coagulopathy, and acidosis. Severe head injury was defined as an Abbreviated Injury Scale (AIS) score ≥ 3 and a GCS score < 8. In addition, AIS is an indicator of the risk of in-hospital mortality and length of ICU stay, especially in patients with severe injuries [41, 42]. All patients were monitored for systemic complications, including inflammation, the duration of mechanical ventilation, and length of stay in the ICU and hospital. The clinical characteristics of the total cohort were divided into two groups: isolated TBI and multiorgan trauma with predominant TBI. The clinical short-term outcome was assessed at hospital discharge using the Glasgow Outcome Scale (GOS) [35] and defined as poor (GOS 1–3) and good (GOS 4–5).

The PSH-AM was used to diagnose PSH. The PSH-AM score has two components: the clinical feature scale, to identify the intensity of cardinal features, and the diagnosis likelihood tool, to evaluate the likelihood of the presence of PSH. Combining the two sums of the total scores yields a total PSH-AM score, which assesses the probability of a PSH diagnosis. A PSH-AM score < 8 indicates the diagnostic probability of PSH as unlikely, a PSH-AM score of 8 to 16 indicates the diagnostic probability of PSH as possible, and a PSH-AM score of more than 17 indicates the diagnostic probability of PSH as likely [43]. A detailed description of the PSH-AM scale is presented in Supplementary Table 1. Based on the clinical course, two independent investigators defined PSH as at least three seizure events with two or more simultaneous features, (1) tachycardia, (2) tachypnea, (3) hypertension, (4) fever, (5) sweating, and (6) sweating dystonia, after excluding other causes (e.g., poorly controlled pain, infection, epileptic seizure).

### Signal Monitoring

The ABP was measured invasively in the radial or femoral artery using a pressure transducer (Argon Standalone DTX Plus, Argon Medical Devices Inc. Plano, TX). ICP was measured invasively using intraparenchymal probes (Codman MicroSensor ICP Transducer, Codman & Shurtleff, Randolph, MA) inserted into the frontal cortex. CPP was defined as the difference between mean ABP and ICP. The cumulative pressure time dosage (PTD; mm Hg/h) was calculated as the area under the curve (AUC) of the ICP or CPP signal above or below the predefined threshold using trapezoidal interpolation [44]. For PTD_ICP_, the upper threshold was set at 22 mm Hg [45], and for PTD_CPP_, the lower threshold was set at 60 mm Hg [39]. Data were digitized with an analog–digital converter and recorded continuously at a sampling frequency of 200 Hz with the Intensive Care Monitor (ICM+) system (Cambridge Enterprise Ltd, Cambridge, UK). Artifacts in the recordings were identified either manually or through custom-written algorithms, and further analyses were performed only on the representative parts of the signals. For patients who required decompression craniectomy, monitoring was discontinued after the surgery. Signal monitoring began within the first 24 h of the patient’s ICU stay and continued throughout their stay. However, for this analysis, we included only the first 5 days of data. The recording period may be shorter than 5 days because of craniectomy or death of the patient. Parameters were estimated in two ways: (1) by calculating an average value from the entire monitoring period (up to 5 days) and (2) by determining daily averages for each metric to observe temporary variations.

### Cerebral Autoregulation

Cerebral autoregulation was characterized using the pressure reactivity index (PRx). The PRx was estimated as the Pearson linear correlation coefficient between slow-wave oscillations of ABP and ICP in a 5-min moving average window updated every 10 s [46] and expressed in an arbitrary unit (a.u.). PRx > 0.3 a.u. was classified as related to ‘impaired’ cerebral autoregulation because it describes more passive transition [47].

### ANS Metrics

BRS was assessed in the time domain based on the sequential cross-correlation method proposed by Westerhof et al. [48]. It was calculated as the slope of the regression line between 10-s segments of the systolic peak-to-peak interval and the corresponding systolic pressure time series derived from the ABP signal. Because of the variability of systolic pressure and interbeat interval, resulting in varying delays between the time series, the algorithm considers time shifts of 0–5 s, and the cross-correlation function is used to obtain the maximum correlation coefficient considering the unknown time shift between the series. An analysis of HRV was performed following the standards described in the Task Force of the European Society of Cardiology and the North American Society of Pacing and Electrophysiology [49]. Because of insufficient quality or unavailability of the electrocardiogram signal in some cases, HRV indices were calculated from the ABP signal using algorithms embedded in the ICM+ software. In the frequency domain, the Lomb–Scargle periodogram [50] was used to determine the power spectral density of the interval time series in the low-frequency range (LF; 0.04–0.15 Hz) and the high-frequency range (HF; 0.15–0.40 Hz). In addition, total power of the HRV signal and the ratio between low- and high-frequency components (LF/HF) were calculated. In the time domain, standard deviation of the sequential beats (SDNN) and the square root of the mean squared differences of successive sequential beats (RMSSD) were determined. A detailed description of the BRS and HRV metrics are presented in Supplementary Table 2.

### Statistics

The normality of data distributions was assessed using the Kolmogorov–Smirnov test with Lilliefors correction. Because of the rejection of the normality hypothesis for most of the analyzed parameters, nonparametric tests were applied. Differences in median values categorized by any dichotomized criteria were tested using the Mann–Whitney *U*-test or Pearson’s *χ*^2^ test (or Fisher’s exact test) for nonnumeric data. To demonstrate the dynamic of ANS parameters in relation to ICP, the total group was divided into quartiles. The Kruskal–Wallis analysis of variance was used to test the differences within those four subgroups. The relationship between ANS metrics and cerebral autoregulation was tested using Spearman’s correlation coefficient. Receiver operating characteristic (ROC) curves were used to determine the predictive power of ANS or neuromonitoring parameters as predictors of PSH syndrome and mortality. Multivariate backward-step logistic regression was employed to develop models of PSH and mortality based on neuromonitoring parameters and ANS metrics found significant in univariate analysis adjusted for clinical risk factors (age, sex, and GCS score for both models, with additional factors such as fever, diffuse axonal injury, and polytrauma for the PSH model). The results of logistic regression were reported as odds ratios (ORs) with corresponding 95% confidence intervals (CI). The statistical significance of the model was assessed using the *χ*^2^ test for the log–likelihood ratio and *χ*^2^ Wald’s test statistics for the factors. The ROC curve with the AUC serving as the evaluation metric was constructed to evaluate the model discriminant ability. A linear mixed-effects model for repeated measures was used to explain daily variations in neuromonitoring, ANS parameters, and cerebral autoregulation with patients as the random effect. The linear mixed-effects model accounts for an unequal number of repeated measurements in our study due to death or craniectomy. Results are presented as median ± IQR unless stated otherwise. The level of significance was set at 0.05. Statistical analyses were conducted using STATISTICA 13 (Tibco, Palo Alto, CA) and R Statistical Software (v.4.0.2; R Foundation for Statistical Computing, Vienna, Austria).

## Results

### Patient Characteristic

Between 2011 and 2022, a total of 528 patients with TBI were admitted to the ICU of the Wroclaw University Hospital and evaluated for inclusion in the study. Of these, 66 patients (27% female) were included. The flowchart is presented in Fig. 1, and clinical characteristics are detailed in Table 1. The median age of the cohort was 33 ± 24 years. The median GCS score on admission was 6 ± 4, with motor subscores averaging 4 ± 2, and the median Injury Severity Scale score was 25 ± 23. Most patients (84%) required circulatory support with vasopressors. Before ICU admission, 15 patients (23%) underwent mass lesion evacuation. Decompressive craniotomy was performed in 16 patients (24%) in whom conservative treatment of ICP was ineffective. Infectious complications occurred in 65% of the total cohort. Seventeen patients (26%) died in the hospital, and irreversible brain damage was the main cause of death. The comparison of clinical characteristics between patients with isolated head trauma (*n* = 23, 35%) and those with polytrauma (*n* = 43, 65%) is presented in Table 1. Patients with polytrauma were younger (29 ± 14 years) than those with isolated brain injury (50 ± 28 years, *p* = 0.001). The main cause of polytrauma was a traffic accident (76%). Additionally, patients with polytrauma more frequently experienced prehospital hypotension (*p* = 0.036), cerebral edema (*p* = 0.020), and diffuse axonal injury (*p* = 0.038) compared to patients with isolated TBI.

**Table 1:** Baseline characteristics of the total cohort of patients with traumatic brain injury categorized by type of injury (isolated head injury or polytrauma with severe head injury)

Characteristic	Total (*N* = 66)	Isolated head injury (*n* = 23)	Polytrauma (*n* = 43)	*p* value
Age, y	33 (26–50)	50 (33–61)	29 (24–38)	**0.001**
Sex (female)	18 (27%)	6 (33%)	12 (67%)	0.874
Cause of injury				
Road traffic incident	37 (56%)	4 (17%)	33 (76%)	**< 0.001**
Incidental fall	4 (6%)	4 (17%)	0	
Other nonintentional injury	18 (27%)	9 (39%)	9 (21%)	
Violence/assault	4 (6%)	4 (17%)	0	
Suicide attempt	1 (2%)	0	1 (2%)	
Other	2 (3%)	2 (10%)	0	
Pupils reactivity				
Bilateral unreactive	27 (41%)	10 (44%)	17 (40%)	0.145
Unilateral unreactive	8 (12%)	5 (22%)	3 (7%)	
Bilateral reactive	31 (47%)	8 (35%)	23 (53%)	
Hypotension (pre-ICU admission)	34 (52%)	8 (35%)	26 (62%)	**0.036**
ISS score	25 (18–41)	16 (16–20)	35 (25–43)	**< 0.001**
GCS at admission	6 (4–8)	6 (5–8)	6 (4–8)	0.385
GCS motor at admission	4 (2–4)	4 (3–5)	4 (2–4)	0.181
CT characteristic				
Marshall CT score	3 (2–4)	3 (2–4)	3 (2–4)	0.860
Rotterdam CT score	3 (2–4)	3 (2–4)	3 (2–4)	0.828
Contusion	36 (54%)	11 (65%)	25 (68%)	0.836
Edema	51 (77%)	14 (28%)	37 (86%)	**0.020**
Subdural hematoma	30 (45%)	11 (48%)	19 (44%)	0.777
Epidural hematoma	7 (11%)	2 (9%)	5 (12%)	0.721
Cerebral hematoma	13 (20%)	7 (30%)	6 (14%)	0.109
Diffuse axonal injury	19 (29%)	3 (13%)	16 (37%)	**0.038**
tSAH	37 (56%)	10 (44%)	27 (63%)	0.132
Other characteristics				
Evacuation of mass lesions	15 (23%)	7 (30%)	8 (19%)	0.278
EVD or CSF drainage	4 (6%)	3 (13%)	1 (2%)	0.082
Decompression craniotomy	16 (24%)	4 (17%)	12 (28%)	0.342
Noradrenaline	56 (84%)	16 (73%)	40 (93%)	**0.025**
MV, days	11 (8–23)	9 (5–16)	13 (8–24)	**0.039**
Epileptic seizures	16 (24%)	7 (30%)	9 (21%)	0.395
Tracheostomy	30 (45%)	11 (48%)	19 (44%)	0.777
Infectious complications	43 (65%)	13 (57%)	30 (70%)	0.281
PSH likely	9 (15%)	3 (13%)	6 (14%)	0.981
PSH possible	13 (20%)	4 (17%)	9 (21%)	0.730
PSH unlikely	44 (65%)	16 (70%)	28 (65%)	0.781
Outcome at hospital discharge				
ICU LOS	21 (12–32)	15 (8–22)	22 (15–35)	**0.008**
Hospital LOS	30 (17–48)	22 (13–36)	33 (20–52)	0.095
Mortality	17 (26%)	7 (42%)	10 (23%)	0.525
GCS	13 (9–14)	14 (11–15)	13 (8–14)	0.239
GOS	3 (2–3)	3 (2–3)	3 (2–3)	0.759

Significant differences are marked in bold. Data are presented as median (upper quartile-lower quartile) or number of study participants (%)

CSF, cerebrospinal fluid; CT, computed tomography; EVD, external ventricular drainage; GCS, Glasgow Coma Scale; GOS; Glasgow Outcome Scale; ICU, intensive care unit; ISS, Injury Severity Scale; LOS, length of stay; MV, mechanical ventilation; PSH, paroxysmal sympathetic hyperactivity; tSAH, traumatic subarachnoid hemorrhage

### Neuromonitoring and ANS Metrics: Daily Variations

**Fig. 2:**
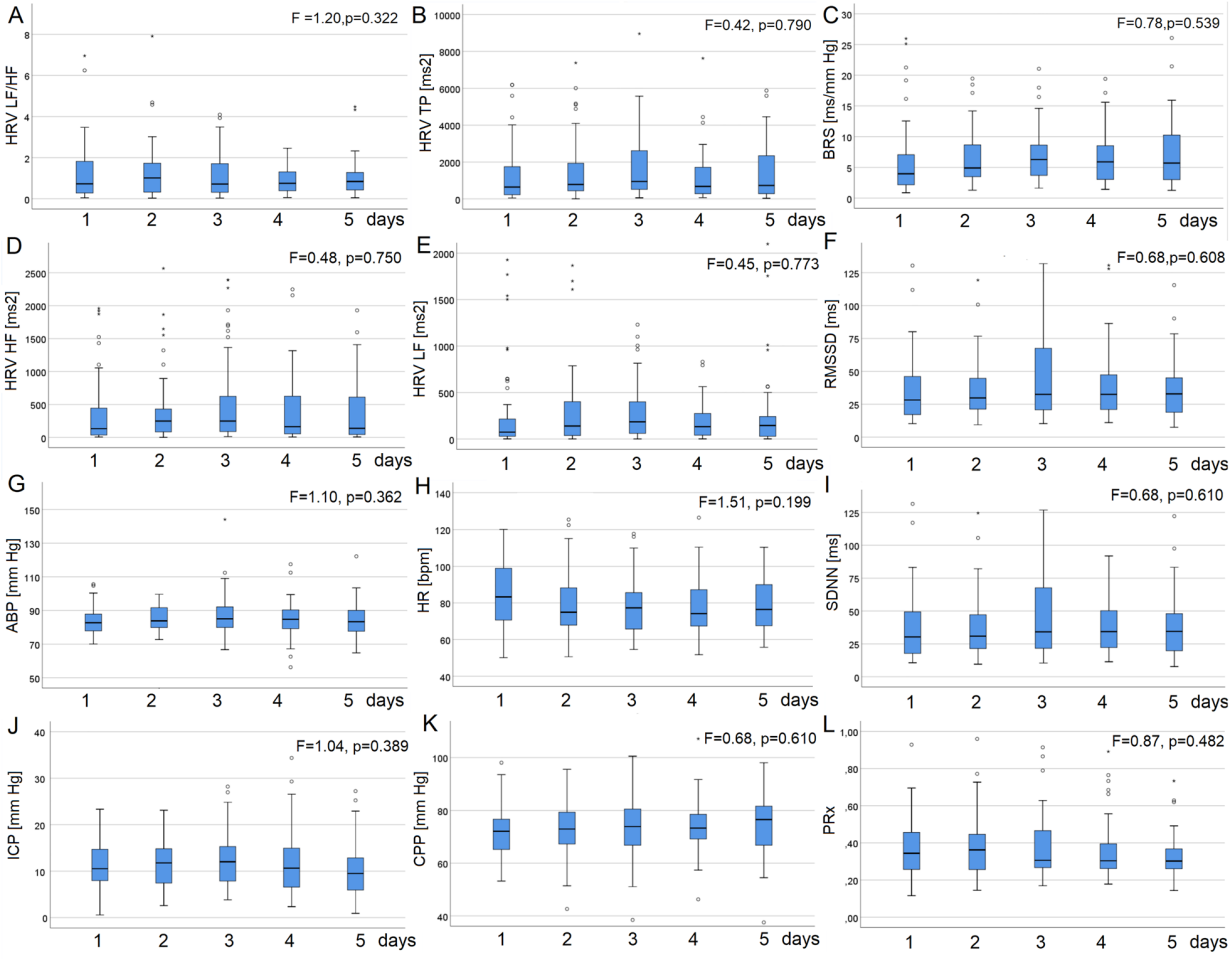
Daily average values for neuromonitoring parameters and autonomic nervous system metrics. No significant changes were found across the cohort from days 1 to 5, using linear mixed-effect model with patients as a random effect. Outliers are marked as circles, and extreme values as asterisks. **a** HRV LF/HF, ratio of low to high frequency in heart rate variability (HRV). **b** TP, total power of HRV. **c** BRS, baroreflex sensitivity. **d** HF, high-frequency range (0.15–0.40 Hz) in HRV. **e** LF, low-frequency range (0.04–0.15 Hz) in HRV. **f** RMSSD, square root of the mean squared differences of successive sequential beats. **g** ABP, arterial blood pressure. **h** HR, heart rate. **i** SDNN, a standard deviation of the sequential beats. **j** ICP, intracranial pressure. **k** CPP, cerebral perfusion pressure. **l** PRx, pressure reactivity index

No significant changes were found in neuromonitoring or ANS metrics in the total cohort using a linear mixed-effects model with patients as a random effect between days 1 and 5. Variations in the daily average of these parameters are presented in Fig. 2. Intracranial hypertension occurred in 16 patients, with an average daily PTD_ICP_ of 8.1 ± 20.4 mm Hg/h. Episodes of low CPP were noted in 18 patients, with an average daily PTD_CPP_ of 7.8 ± 28.6 mm Hg/h.

### Neuromonitoring and ANS Metrics: Average Values

The median values of neuromonitoring parameters ANS metrics and cerebral autoregulation for the total group of patients, as well as by the type of trauma (polytrauma vs. isolated head injury), are presented in Table 2. Compared to patients with polytrauma, those with isolated injury had higher SDNN (49 ± 44 vs. 34 ± 32 ms; *p* = 0.046) and higher RMSSD (45 ± 42 vs. 33 ± 30 ms; *p* = 0.041). Other parameters did not show significant differences related to the type of injury (see Table 2). The results of the ANS–ICP relationship are detailed in the Supplementary Data, which indicate that the ANS–ICP relationship is nonlinear, with ICP having the strongest impact on HRV LF/HF (*p* = 0.004).

**Table 2:** Neuromonitoring parameters, cerebral autoregulation, and autonomic nervous system metrics vs. type of injury (isolated head injury vs. polytrauma), diagnosis of PSH syndrome (likely vs possible/unlikely), in-hospital mortality (nonsurvivors vs survivors) and short-term outcome (good vs poor) in patients with traumatic brain injury.

Characteristic	Total (*N* = 66)	Short-term outcome		In-hospital mortality		PSH		Type of injury	
		poor outcome (*n* = 39)	good outcome (*n* = 27)	Nonsurvivors (*n* = 17)	Survivors (*n* = 49)	Likely (*n* = 9)	Possible or unlike (*n* = 57)	Isolated head injury (*n* = 23)	Polytrauma (*n* = 43)
ABP, mm Hg	84 ± 10	85 ± 14	84 ± 7	80 ± 14	85 ± 9	88 ± 10	84 ± 10	89 ± 14	83 ± 8
HR, bpm	77 ± 19	76 ± 23	79 ± 17	74 ± 23	78 ± 18	70 ± 7	78 ± 19*	73 ± 18	77 ± 20
CPP, mm Hg	73 ± 13	75 ± 17	73 ± 10	67 ± 22	75 ± 9	75 ± 23	73 ± 11	75 ± 14	72 ± 11
pCO_2_, kPa	36.0 ± 2.4	36.0 ± 2.8	36.0 ± 2.9	36.5 ± 3.4	36.0 ± 2.0	35.6 ± 0.9	36.2 ± 2.6	35.6 ± 0.9	36.2 ± 2.6
ICP, mm Hg	12 ± 7	12 ± 9	12 ± 6	14 ± 10	12 ± 5	10 ± 14	12 ± 8	36.0 ± 2.0	12 ± 8
PRx, a.u	0.35 ± 0.15	0.35 ± 0.19	0.35 ± 0.13	0.37 ± 0.20	0.34 ± 0.12*	0.32 ± 0.10	0.36 ± 0.14	0.33 ± 0.11	0.36 ± 0.16
RMSSD, ms	36 ± 30	37 ± 34	33 ± 25	49 ± 38	33 ± 27*	34 ± 13	36 ± 32	45 ± 42	33 ± 30*
SDNN, ms	38 ± 35	39 ± 39	34 ± 27	53 ± 41	34 ± 29*	37 ± 15	37 ± 34	49 ± 44	34 ± 32*
HRV LF, Hz	211 ± 301	228 ± 225	148 ± 256	233 ± 145	176 ± 274	253 ± 178	176 ± 227*	233 ± 452	195 ± 296
HRV HF, Hz	295 ± 537	394 ± 832	203 ± 428	527 ± 958	240 ± 479	489 ± 380	232 ± 526	349 ± 613	257 ± 516
HRV TP, Hz	1,290 ± 1,360	1,330 ± 1,318	1029 ± 1,423	1,641 ± 1,024	1,121 ± 1,315	1,328 ± 379	1,177 ± 1,602	1,365 ± 1,685	1,122 ± 1,587
HRV LF/HF, a.u	0.85 ± 1.20	0.82 ± 1.26	0.93 ± 1.15	0.32 ± 0.63	0.99 ± 1.12**	0.81 ± 0.19	0.90 ± 1.25	0.95 ± 0.95	0.76 ± 1.33
BRS, ms/mm Hg	6.4 ± 4.8	6.3 ± 4.7	6.5 ± 5.5	7.5 ± 5.6	6.3 ± 4.7	6.8 ± 2.2	6.0 ± 5.3	6.9 ± 5.4	5.4 ± 4.7

Data are presented as median ± IQR

ABP, arterial blood pressure; a.u., arbitrary unit; BRS, baroreflex sensitivity; CPP, cerebral perfusion pressure; GOS, Glasgow Outcome Scale; HF, high-frequency range (0.15–0.40 Hz); HR, heart rate; HRV, heart rate variability; ICP, intracranial pressure; LF, low-frequency range (0.04–0.15 Hz); LF/HF, low-to-high-frequency range; pCO_2_, partial carbon dioxide concentration; PRx, pressure reactivity index; PSH, paroxysmal sympathetic hyperactivity; RMSSD, square root of the mean squared differences of successive sequential beats; SDNN, a standard deviation of the sequential beats; TP, total power

*p < 0.05; **p < 0.01

### Relationship Between ANS and Cerebral Autoregulation

**Fig. 3:**
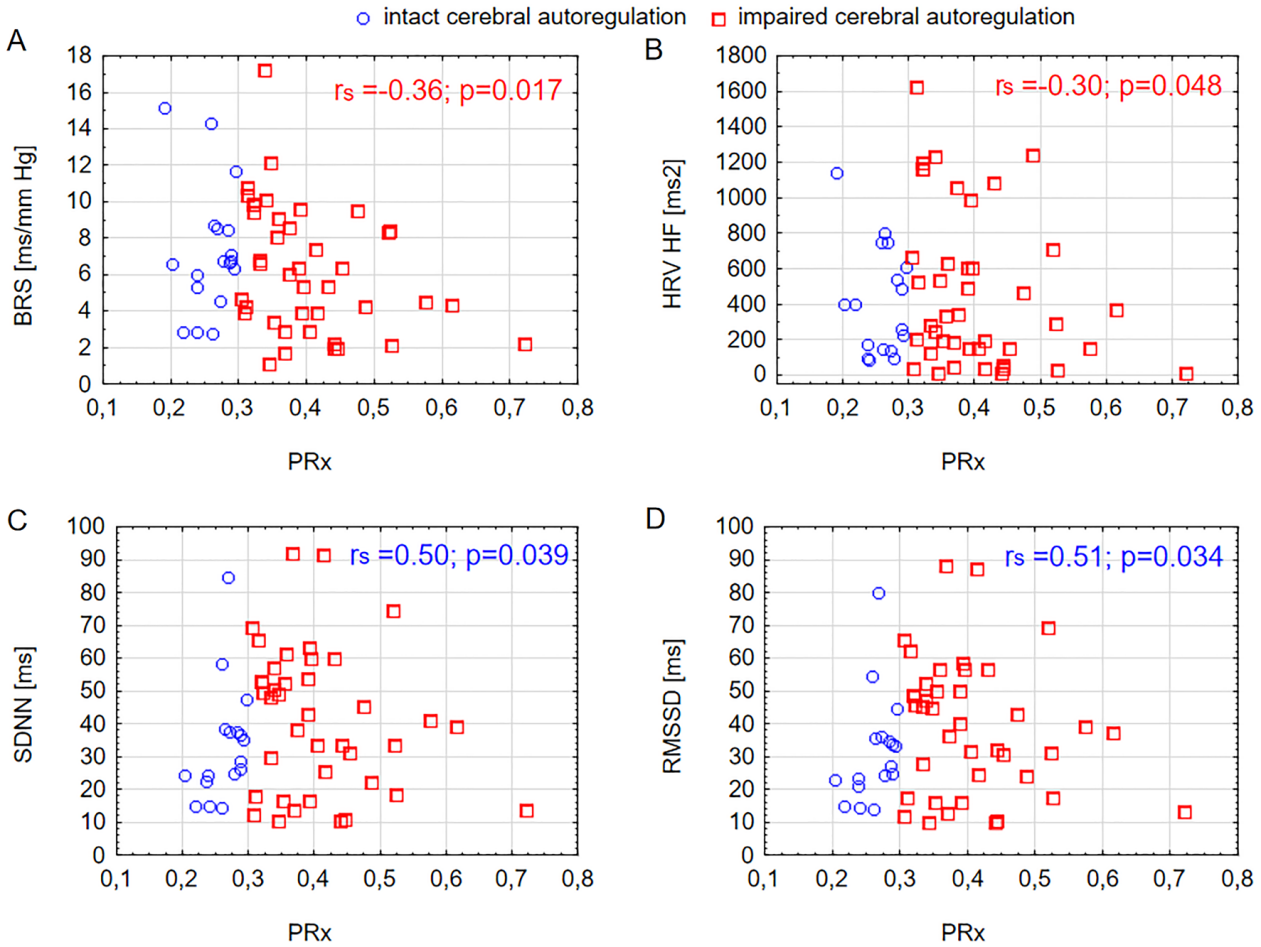
Spearman correlation (r_S_) between cerebral autoregulation (PRx) and **a** Baroreflex sensitivity (BRS). **b** Heart rate variability (HRV) in the high-frequency range (HF, 0.15–0.40 Hz). **c** Standard deviation of the sequential beats (SDNN). **d** Square root of the mean squared differences of successive sequential beats (RMSSD) in patients with intact cerebral autoregulation (PRx < 0.3) and impaired cerebral autoregulation (PRx > 0.3) (Color figure online)

Impaired cerebral autoregulation was found in 48 patients (73%). Within this group, a moderate correlation was observed between poorer cerebral autoregulation (higher PRx) and lower BRS (*r*_S_ =  − 0.36, *p* = 0.017), as shown in Fig. 3a. Additionally, a moderate correlation was found between worse cerebral autoregulation and lower HRV in the HF (*r*_S_ =  − 0.30, *p* = 0.048), as illustrated in Fig. 3b. In patients with intact cerebral autoregulation, there was a reciprocal relationship between PRx and HRV SDNN (*r*_S_ = 0.50, *p* = 0.039; see Fig. 3c) and HRV RMSSD (*r*_S_ = 0.51, *p* = 0.034; see Fig. 3d).

### PSH Syndrome

PSH syndrome was classified as likely in 9 (14%) patients, possible in 13 (20%), and unlikely in 44 (66%). This classification was based on clinical indicators assessed between days 9 and 15 of ICU treatment and thus was made after the period of signals analysis (we included only the first 5 days). The comparison of clinical risk factors for PSH between the likely and possible/unlikely groups is presented in Supplementary Table 3. Notably, except for tracheostomy (*p* < 0.001) and diffuse axonal injury (*p* = 0.013), other clinical parameters (age, fever, or polytrauma) were not significant indicators. All nine patients with PSH had poor outcomes. Among these, six patients (67%) had impaired cerebral autoregulation; however, this ratio is comparable to that of patients with possible/unlikely PSH (41 of 57, 72%). The comparison of median values of neuromonitoring parameters, ANS metrics, and cerebral autoregulation in patients with PSH and those with possible/unlikely PSH is shown in Table 2. Patients with PSH had a lower HR (70 ± 7 bpm) compared to those without PSH (78 ± 19 bpm, *p* = 0.027). Additionally, patients with PSH had higher HRV in the LF (253 ± 178 ms^2^) than those without PSH (176 ± 227 ms^2^; *p* = 0.035). There was no significant difference in the occurrence of periods of intracranial hypertension or too low CPP between patients with PSH (22% and 22%, respectively) and those without PSH (25% and 29%, respectively).

### Prediction of PSH

**Fig. 4:**
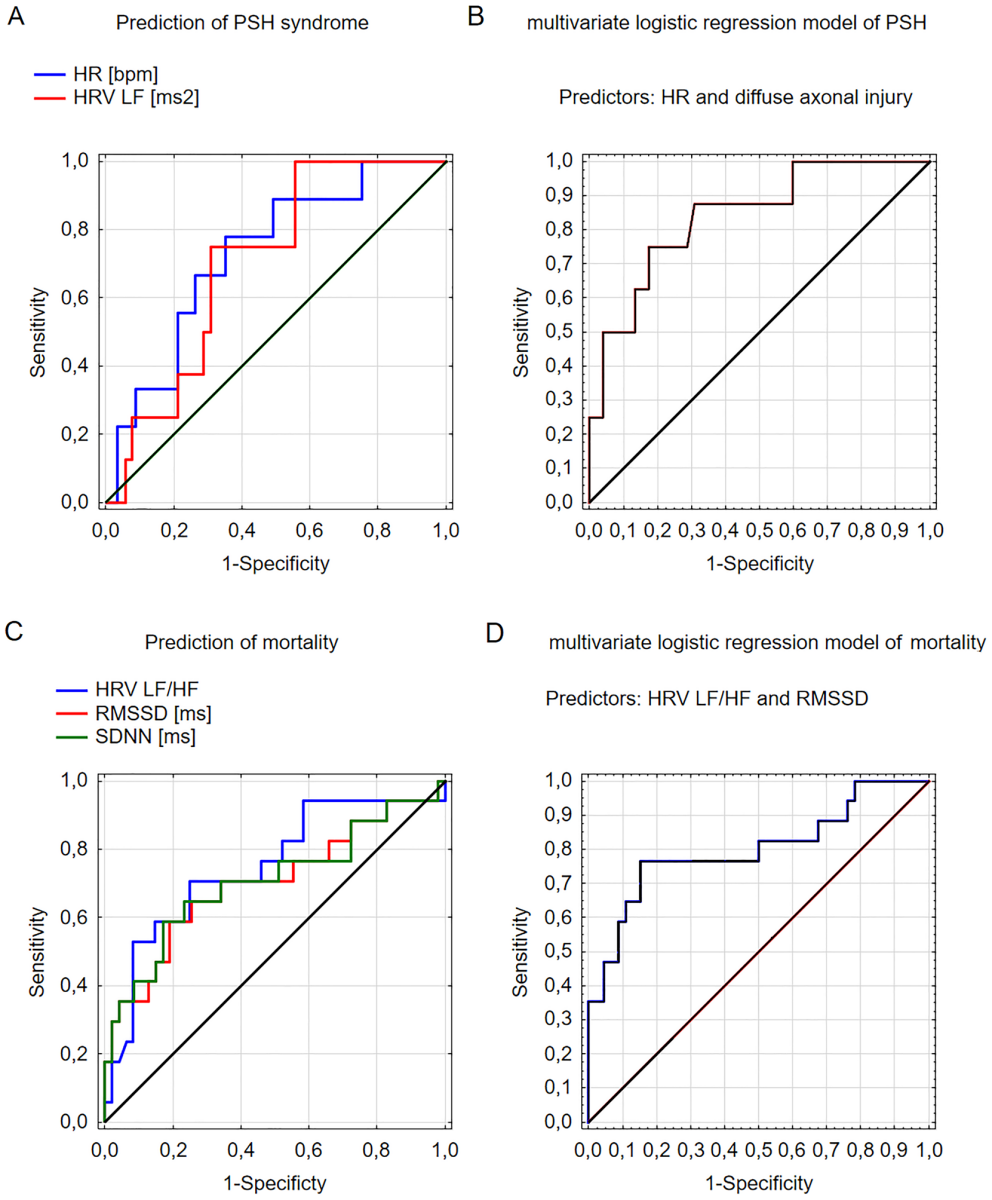
(Upper panel) Receiver operating characteristic (ROC) curves for predicting paroxysmal sympathetic hyperactivity (PSH) syndrome using independent predictors: heart rate (HR) and the low-frequency part of a spectrum of heart rate variability (0.04–0.15 Hz) (HRV LF) (**a**). **b** Multivariate logistic regression model including two significant factors: diffuse axonal injury and heart rate (HR). (Lower panel) ROC curves for predicting mortality using independent predictors: RMSSD, square root of the mean squared differences of successive sequential beats; SDNN, a standard deviation of the sequential beats; HRV LF/HF, low-to-high-frequency range of heart rate variability (**c**). **d** Multivariate logistic regression model including two significant factors: RMSSD and HRV LF/HF

A ROC curve analysis indicated that both HR (AUC = 0.73, *p* = 0.006) and HRV in the LF (AUC = 0.70, *p* = 0.009) are moderate predictors of PSH, as shown in Fig. 4a. In the multiple logistic regression model for PSH, HRV in the LF and HR were adjusted by standard clinical factors (age, sex, GCS score) and risk factors for PSH syndrome (fever, diffuse axonal injury, polytrauma). The final model (*χ*^2^ = 8.59, *p* = 0.014) is detailed in Table 3. It included diffuse axonal trauma (OR 10.82, 95% CI 1.70–68.98, *p* = 0.012) and HR (OR 0.91, 95% CI 0.84–0.98, *p* = 0.021). The model demonstrated moderate prediction ability (AUC = 0.84), as illustrated in Fig. 4b.

**Table 3:** Logistic regression model of paroxysmal sympathetic hyperactivity (PSH) and mortality in the total cohort of patients with traumatic brain injury

Variable	*β*	OR	95% CI	*p* value
PSH model				
Diffuse axonal injury				
Absent (ref.)		1		
Present	2.38	10.82	1.70–68.98	0.012
HR (bpm)	− 0.10	0.91	0.84–0.98	0.021
Mortality model				
HRV LF/HF	− 1.45	0.23	0.07–0.75	0.014
RMSSD (ms)	0.05	1.05	1.01–1.08	0.006

*β*, regression coefficient; CI, confidence interval; HR, heart rate; HRV LF/HF, low-to-high-frequency range of heart rate variability; OR, odds ratio; PSH, paroxysmal sympathetic hyperactivity; RMSSD, square root of the mean squared differences of successive sequential beats

### Prediction of Mortality

Clinical characteristics versus short-term outcomes are presented in Supplementary Table 4. No significant differences were observed in neuromonitoring parameters, ANS metrics, and cerebral autoregulation regarding short-term outcome (see Table 2). In patients who died, the LF/HF was significantly lower compared to that in survivors (0.32 ± 0.63 vs. 0.99 ± 1.12; *p* = 0.003), whereas RMSSD and SDNN were significantly higher (49 ± 38 vs. 33 ± 27 ms [*p* = 0.015] and 53 ± 41 vs. 34 ± 29 ms [*p* = 0.013]), as shown in Table 2. Additionally, patients who died had worse cerebral autoregulation, indicated by higher PRx (0.37 ± 0.20 vs. 0.34 ± 0.12 a.u., *p* = 0.042). The ROC curve analysis showed that LF/HF (AUC = 0.75, *p* = 0.001), RMSSD (AUC = 0.70, *p* = 0.015), and SDNN (AUC = 0.71, *p* = 0.013) were independent predictors of mortality (see Fig. 4a). In the multiple logistic regression model for mortality, HRV LF/HF, SDNN, and RMSSD were adjusted by standard clinical factors (age, sex, GCS score). The final model (*χ*^2^ = 10.72, *p* = 0.004) is presented in Table 3. It included HRV LF/HF (OR 0.23, 95% CI 0.07–0.75, *p* = 0.014) and RMSSD (OR 1.05, 95% CI 1.01–1.08, *p* = 0.006). This model demonstrated moderate prediction ability (AUC = 0.80; see Fig. 4b).

## Discussion

Our study demonstrated that ANS disorders in the early stages of TBI are associated with the development of PSH syndrome. To the best of our knowledge, this study is among the first to evaluate the utility of HRV assessment and cerebral autoregulation based on continuous monitoring in acute TBI for the early prediction of PSH syndrome. We found that a high HRV in the LF, reflecting modulation of cardiac autonomic outflows by baroreflexes [51], and a low HR were moderate independent early predictors of PSH development. In the adjusted model of PSH, we found that HR and diffuse axonal injury emerged as significant factors influencing the development of PSH. Notably, standard clinical risk factors, such as fever, age, and polytrauma, were not important predictors. Additionally, we found that a decreased LF/HF, along with higher RMSSD and SDNN, contributed to an increased risk of death, underscoring the importance of ANS monitoring in patients with TBI. Although we noted impairment in cerebral autoregulation in a comparable ratio both in patients with PSH and in patients with possible/unlikely PSH, the impact of those regulatory mechanisms requires further studies.

Our study showed that changes in ANS modulation after severe TBI are related to the type of injury. In patients with isolated head injury, SDNN and RMSSD were higher than those in patients with multiple trauma, despite the age differences; patients with polytrauma were significantly younger than patients with isolated head injury. According to clinical guidelines, RMSSD mainly reflects parasympathetic activity mediated by the vagus nerve [49]. SDNN reflects both parasympathetic and sympathetic activity, and its low value is related to higher mortality and morbidity [52].

Previous studies have shown that TBI leads to the development of ANS abnormalities [53–55]. During the acute phase of the injury, the initial increase in ICP triggers the activation of the sympathetic nervous system, resulting in elevated circulating catecholamines, increased blood pressure, increased HR, increased cardiac output, and heightened vascular resistance [53]. Following this hyperdynamic phase, there is a subsequent decrease in circulating catecholamines and vascular resistance, leading to hypotension and impaired organ perfusion. This impairment of hemodynamic stability, coupled with impaired autoregulation of the cerebral circulation reflecting the influence of impaired autonomic system control on cerebral vessels, can contribute to secondary brain damage [56, 57]. Dysautonomia after TBI depends on the location and extent of both primary and secondary brain injuries [9]. One of the symptoms of ANS disorders is PSH syndrome, which can manifest at any point during TBI but most commonly within 2 weeks of onset [10, 58]. The diagnosis of PSH relies on excluding other pathological conditions with a similar clinical presentation, such as epileptic seizures, pulmonary embolism, or sepsis. Moreover, the ANS is influenced by the therapeutic interventions themselves, including mechanical ventilation, sedation, analgesics, vasopressors, and inotropic drugs. That is why early diagnosis of PSH is difficult and prediction models are needed.

Several studies highlight the clinical burden of PSH, with potential complications such as morbidity, infections, weight loss, dehydration, disruption of rehabilitation, and prolonged ICU stay [21, 22]. Uncontrolled symptoms may contribute to secondary brain damage through hypertension, hyperthermia, cardiac damage, and even mortality [58]. The most widely accepted pathophysiological theory of PSH syndrome is the excitatory/inhibitory ratio, which suggests that injury impairs descending inhibition from the cortex, thalamus, and hypothalamus, leading to the development of excitatory spinal circuits [21]. Consequently, stimuli that are either non-nociceptive (movement) or minimally nociceptive can trigger excitation of spinal circuits, followed by cessation of contractions due to restoration of inhibitory factors [43, 59]. Additionally, changes observed during a seizure include dysfunction of neurotransmitters (glutamate and γ-aminobutyric acid) and their receptors, the release of excitatory amino acids, and activation of the hypothalamic–pituitary–adrenal axis with the uncontrolled release of catecholamines (and possibly corticosteroids) [60, 61]. Furthermore, secondary brain damage due to the production of inflammatory cytokines and reactive oxygen species, as well as general deterioration, may be associated with PSH [62]. Risk factors for PSH include younger age, worse GCS score, tracheostomy, diffuse TBI, and polytrauma [21, 62]. In our study, PSH syndrome was diagnosed between 9 and 15 days of treatment, irrespective of the type of injury (isolated or polytrauma), and age and fever were not significant predictors. Statistically significant differences were found in the occurrence of diffuse axonal injury and tracheostomy between the group with PSH and the group without PSH. A study by Hinson et al. [63] demonstrated that in addition to these risk factors, early fever occurring within 24 h of onset is a predictor of PSH development. Most of our patients had a fever within the first 48 h of follow-up, negative bacteriological test results, and no clinical signs of infection. Thus, the challenges regarding PSH syndrome include not only making the correct diagnosis, which has been significantly improved by the definition introduced in 2014 [64], but also correctly stratifying the risk of developing PSH.

Impairment in cerebral autoregulation has been reported as an independent predictor of mortality in acute brain injury [65–69], and changes in cerebral autoregulation, along with alterations in serum biomarkers levels, may predict in cerebral ischemia [70]. However, the interplay between ANS disorders and disruptions in cerebral autoregulation is a subject of debate [68]. Previous studies on patients with TBI have either focused on abnormal autoregulation of cerebral circulation [68, 71–73] or analyzed HRV metrics during the acute phase of TBI in relation to treatment outcomes [9, 16, 17, 74, 75]. In our analysis, we explored the relationship between cerebral autoregulation and ANS metrics. Interestingly, we found that in pathological conditions when the cerebral autoregulatory mechanism is exhausted (PRx > 0.3 a.u.), worse cerebral autoregulation is associated with lower BRS and lower HRV in the HF (a measure of efferent vagal activity) [52]. Conversely, when cerebral autoregulation is preserved, the correlation between autoregulation and ANS metrics is reciprocal; higher PRx correlates with higher RMSSD or SDNN. A previous study by Lavinio et al. [16] suggested that the loss of cerebral autoregulation following TBI might be a manifestation of acute autonomic failure. They reported a moderate correlation between PRx and HRV in the HF in 18 patients with severe TBI, indicating that an HRV analysis could become a practical tool for screening patients at risk of cerebral autoregulation derangement. Froese et al. [54] demonstrated that disturbances in cerebral autoregulation in patients with TBI could potentially lead to variations in systemic circulatory autonomic responses. However, in some patients, fluctuations in ANS responses result in more pronounced changes in cerebral autoregulation compared to the reverse situation. These findings emphasize the complex interplay between autonomic function and cerebral hemodynamic in the context of TBI. In this study, we did not analyze the impact of the cerebral autoregulation–ANS relationship on PSH syndrome development because of a limited number of patients with this syndrome. However, further investigation is required to understand the prognostic impact of this relationship in patients with TBI.

It is known that autonomic disorders significantly affect outcome after brain trauma [17, 53, 76]. However, we found no significant difference in neuromonitoring parameters or ANS metrics regarding short-term outcome. Nevertheless, we found that anisocoria, lower GCS score on admission, higher Marshall score, presence of diffuse axonal injury, seizures, and occurrence of PSH syndrome had a significant impact on outcome. This is in line with previous studies that have shown that extracranial injuries in TBI may modify functional outcomes and increase mortality risk [77–79]. In the total cohort of patients, there was a significant association between decreased LF/HF, reflected imbalance between sympathetic and parasympathetic branches of the ANS, and in-hospital mortality. However, the utility of LF/HF is controversial, as demonstrated by Billman [80], who noted that its utility as “sympatho-vagal balance” depends on different interrelated assumptions and is confounded by the mechanical effects of respiration and prevailing HR.

In our study, we did not observe any significant changes in the daily averages of ANS parameters. However, analyzing temporary patterns and daily variations in the ANS seems to be a promising approach [64]. Previous studies have shown that even short episodes of hypotension or a suboptimal CPP level can significantly impact neurological outcome, thus offering valuable prognostic information [81]. These episodes might be blurred when averaging data over the total monitoring period. Notably, such episodes could be better characterized using daily PTD, a metric employed in the current research to track intracranial hypertension events [82]. In this study, we did not find a significant difference in the occurrence of intracranial hypertension or low CPP between patients with PSH and those without PSH. However, further research is needed to investigate the prognostic potential of these episodes.

This study has limitations. TBI may disrupt the autonomic system’s response to shock, particularly in cases of polytrauma [83, 84]. Although our study group consisted of hemodynamically stable patients, this aspect should be acknowledged. In our analysis, we used data collected from patients who were hospitalized several years ago. Nevertheless, patients’ management followed standardized protocols that were close to the recently published treatment guidelines [38, 39]. Our cohort of patients was homogeneous in terms of mechanical ventilation and medications. The majority of the group was under the influence of vasopressors, with propofol being the main sedative. It should be noted although recent studies have shown that the supply of sedatives, analgesics, and vasopressors has no clinically significant effect on cerebral vascular reactivity [85, 86], their impact is still under investigation . The limited number of study participants in the subgroup analysis may introduce bias and reduce the statistical power of the test. Therefore, these results need to be confirmed in a large multicenter study.

## Conclusions

Elevated HRV in the LF and decreased HR may be used as an early predictor of PSH syndrome development, especially in patients with diffuse axonal injury. Further research is required to investigate the utility of the cerebral autoregulation–ANS relationship in PSH prognostication.

## Supplementary Information

Below is the link to the electronic supplementary material.

Supplementary file1 (DOCX 220 kb)
