# Supplementary material for: Heart Rate Variability and Cerebral Autoregulation in Patients with Traumatic Brain Injury with Paroxysmal Sympathetic Hyperactivity Syndrome
Source: Neurocrit Care. 2024 Oct 29;42(3):864–77. doi: 10.1007/s12028-024-02149-1 (PMC12137448; doi:10.1007/s12028-024-02149-1)
Supplement: Supplementary file 1 — Supplementary file1 (DOCX 220 kb) [file 12028_2024_2149_MOESM1_ESM.docx]

**Supplementary Data**

**ANS metrics and ICP**

We observed that the ANS-ICP characteristic is non-linear. HRV LF was the lowest when ICP was below the lower quartile (< 7 mm Hg), and then from the median to the upper quartile, we observed a decrease in its value (ANOVA p = 0.048, Supplementary Figure 1 A). HRV HF significantly decreased with ICP (p = 0.049, Supplementary Figure 1 B). HRV LF/HF increased non-linearly with ICP
(p = 0.009) until the upper breakpoint at a mean ICP of 14 mm Hg (Supplementary Figure 1 C). The relationship between HRV TP, BRS, RMSSD, and SDNN with ICP was not significant.

**
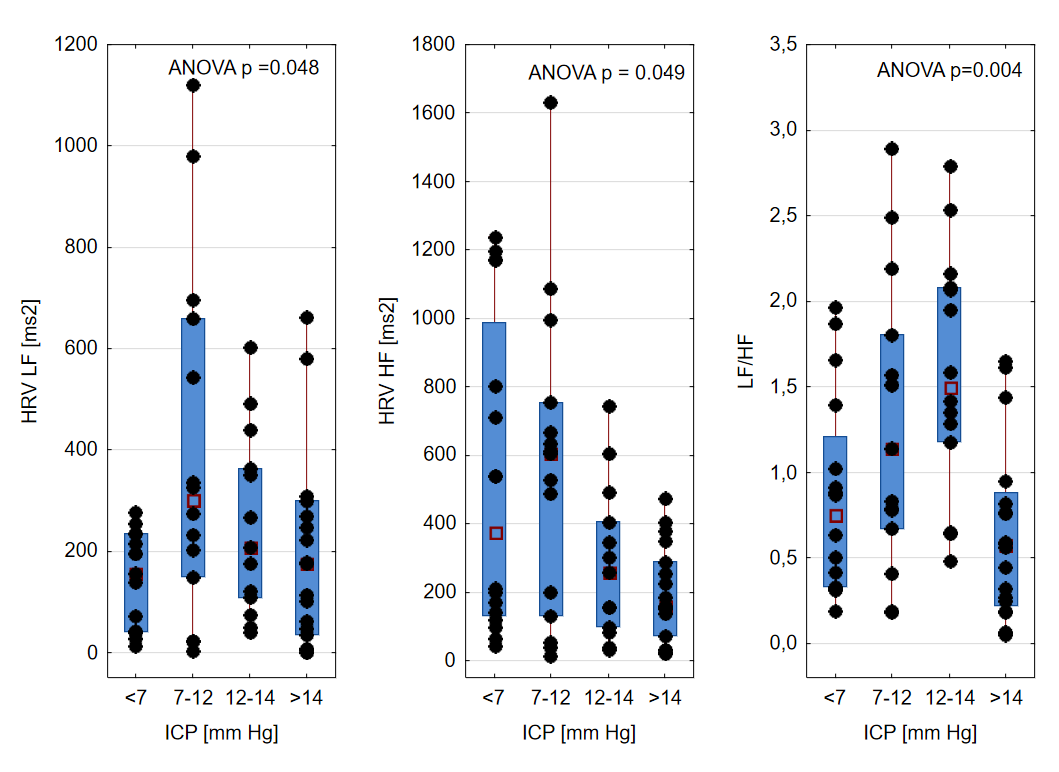
**

**Supplementary Figure 1**

Relationship between autonomic nervous system metrics (ANS): **A**. heart rate variability (HRV) in the low-frequency range (0.04-0.15 Hz), **B**. HRV in the high-frequency range (0.15-0.40 Hz), **C**. HRV low-frequency to high-frequency (LF/HF) ratio versus intracranial pressure (ICP), where ICP is divided into quartiles. Data are shown as median values with interquartile ranges (boxes), and minimum-maximum values (ends of whiskers).

**Supplementary Table 1.**

Paroxysmal sympathetic hyperactivity (PSH) assessment measure according to [1] [2].

| **Clinical Feature Scale (CSF)** | | | | | |
| --- | --- | --- | --- | --- | --- |
|  | 0 | 1 | 2 | 3 | Score |
| Hear rate | <100 | 100-119 | 120-139 | >=140 | 2 |
| Respiratory rate | <18 | 18-23 | 24-29 | >= 30 | 2 |
| Systolic blood pressure | <140 | 140-159 | 160-179 | >= 180 | 3 |
| Temperature | <37 | 37-37.9 | 38-38.9 | >= 39 | 2 |
| Sweating | Nil | Mild | Moderate | Severe | 3 |
| Posturing during episodes | Nil | Mild | Moderate | Severe | 1 |
| **CSF subtotal** | | | | | |
| **Diagnosis Likelihood Tool (DLT) (Score 1 point for each feature present)** | | | | | |
| Clinical features occur simultaneously | | | | | |
| Episodes are paroxysmal in nature | | | | | |
| Sympathetic over-reactivity to normally non-painful stimuli | | | | | |
| Features persist >= 3 consecutive days | | | | | |
| Features persist >= 2 weeks post brain injury | | | | | |
| Features persist despite treatment of alternative differential diagnosis | | | | | |
| Medication administered to decrease sympathetic features | | | | | |
| >= 2 episodes daily | | | | | |
| Absence of parasympathetic features during episodes | | | | | |
| Absence of other presumed cause of features | | | | | |
| Antecedent-acquired brain injury | | | | | |
| **DLT subtotal** | | | | | |
| **Combined total (CSF + DLT)** | | | | | |
| **PSH diagnostic likelihood** | | | Unlikely | <8 | |
|  |  |  | Possible | 8-16 | |
|  |  |  | Probable | >17 | |

[1] Baguley IJ, Perkes IE, Fernandez-Ortega J-F, Rabinstein AA, Dolce G, Hendricks HT, for the Consensus Working Group. Paroxysmal sympathetic hyperactivity after acquired brain injury: consensus on conceptual definition, nomenclature, and diagnostic criteria. J Neurotrauma 2014;31:1515–1520.

[2] Monteiro FB, Fonseca RC, Mendes R. Paroxysmal Sympathetic Hyperactivity: An Old but Unrecognized Condition. Eur J Case Rep Intern Med. 2017 Apr 27;4(3):000562.

**Supplementary Table 2.** Heart rate variability (HRV) indices, baroreflex sensitivity (BRS), and their interpretation.

| **Variable** | **Definition** | **Interpretation** |
| --- | --- | --- |
| BRS  (ms/mm Hg) | Baroreflex sensitivity | It quantifies the relationship between heart rate (HR) and changes in arterial blood pressure. It provides a useful synthetic index of neural regulation at the sinus atrial node (Kazimierska et al., 2019; La Rovere et al., 2008) |
| **Time domain** | | |
| SDNN  (ms) | The standard deviation of normal-to-normal beat intervals. | The standard deviation of NN (SDNN) reflects the parasympathetic component of the autonomic function. It reflects the decrease in SDNN, decreased vagal activity and increased sympathetic activity of the sinus node (Burr, 2007; Pinna et al., 2015). |
| RMSSD  (ms) | Root mean square of successive differences, between normal-to-normal beat intervals. | The RMSSD reflects the beat-to-beat variance in HR and is the primary time-domain measure used to estimate the vagally mediated changes reflected in HRV. RMSSD is correlated with HF power, and influenced by the parasympathetic nervous system (PNS) (Shaffer & Ginsberg, 2017). |
| **Frequency domain** | | |
| LF  (ms^2^) | Low-frequency power;  0.04-0.15Hz | Low-frequency power is modulated by baroreflexes with a combination of sympathetic and parasympathetic efferent nerve traffic to the sinoatrial node (Burr, 2007; Galletly & Larsen, 2001). |
| HF  (ms^2^) | High-frequency power;  0.15-0.4Hz | It is modulated by the parasympathetic activity of ANS and that is the major determinant of respiratory sinus arrhythmia (Berntson et al., 1997). |
| LF/HF | The ratio of LF over HF | It reflects a general balance between the sympathetic-to-parasympathetic branch, according to the assumption that LF power may be generated by the SNS while HF power is produced by the PNS. However, its interpretation is still the matter of debate (Billman, 2013). |
| TP  (ms^2^) | Total power | Reflects all components of the autonomic regulation and is a measure of total variability (Shaffer et al., 2014). |

**References**

Berntson, G. G., Bigger, J. T., Eckberg, D. L., Grossman, P., Kaufmann, P. G., Malik, M., Nagaraja, H. N., Porges, S. W., Saul, J. P., Stone, P. H., & van der Molen, M. W. (1997). HRV_Orgins, methods, and interpretive caveats. *Psychophysiology*, *34*, 623–648. https://doi.org/10.1111/j.1469-8986.1997.tb02140.x

Billman, G. E. (2013). The LF/HF ratio does not accurately measure cardiac sympatho-vagal balance. *Frontiers in Physiology*, *4 FEB*, 45733. https://doi.org/10.3389/FPHYS.2013.00026/BIBTEX

Burr, R. L. (2007). Interpretation of normalized spectral heart rate variability indices in sleep research: a critical review. *Sleep*, *30*(7), 913–919. https://doi.org/17682663

Galletly, D. C., & Larsen, P. D. (2001). Cardioventilatory coupling in heart rate variability: methods for qualitative and quantitative determination. *Br J Anaesth*, *87*(6), 827–833. https://doi.org/10.1093/bja/87.6.827

Kazimierska, A., Placek, M. M., Uryga, A., Wachel, P., Burzyńska, M., & Kasprowicz, M. (2019). Assessment of Baroreflex Sensitivity Using Time-Frequency Analysis during Postural Change and Hypercapnia. *Computational and Mathematical Methods in Medicine*, *2019*. https://doi.org/10.1155/2019/4875231

La Rovere, M. T., Pinna, G. D., & Raczak, G. (2008). Baroreflex sensitivity: measurement and clinical implications. *Annals of Noninvasive Electrocardiology*, *13*(2), 191–207. https://doi.org/10.1111/j.1542-474X.2008.00219.x

Pinna, G. D., Maestri, R., & La Rovere, M. T. (2015). Assessment of baroreflex sensitivity from spontaneous oscillations of blood pressure and heart rate: Proven clinical value? *Physiological Measurement*, *36*(4), 741–753. https://doi.org/10.1088/0967-3334/36/4/741

Shaffer, F., & Ginsberg, J. P. (2017). An Overview of Heart Rate Variability Metrics and Norms. *Frontiers in Public Health*, *5*, 258. https://doi.org/10.3389/FPUBH.2017.00258

Shaffer, F., Mccraty, R., Zerr, C. L., & Medical, D. V. a. (2014). A healthy heart is not a metronome: an integrative review of the heart ’ s anatomy and heart rate variability. *Frontiers in Psychology*, *5*, 1–19. https://doi.org/10.3389/fpsyg.2014.01040

**Supplementary Table 3.** The comparison of clinical risk factors for paroxysmal hyperactivity (PSH) between the group of patients who developed PSH (likely) and those who did not (possible or unlikely). Data are presented as median ± interquartile range or number of observations (% of the group).

| Characteristic | PSH  likely  n = 9 | PSH  possible or unlikely  n = 57 | p-value |
| --- | --- | --- | --- |
| Age [years] | 43±46 | 33±19 | 0.385 |
| Diffuse axonal injury | 6 (67%) | 13 (23%) | **0.013** |
| Fever | 6 (67%) | 40 (70%) | 0.555 |
| Tracheostomy | 9 (100%) | 21 (39%) | **< 0.001** |
| Polytrauma | 6 (67%) | 37 (65%) | 0.617 |

**Supplementary Table 4**. Baseline characteristics of the total cohort of patients with traumatic brain injury (TBI) versus short-term outcome in Glasgow Outcome Scale (GOS). Significant differences are marked in bold. Data are presented as median ± interquartile range or number of subjects (%).

| Characteristic | Total  N = 66 | GOS 1-3  n = 39 | GOS 4-5  n = 27 | p-value |
| --- | --- | --- | --- | --- |
| Age | 33 (26-50) | 37 (27-62) | 30 (24-38) | **0.039** |
| Female | 18 (27%) | 14 (36%) | 4 (15%) | 0.051 |
| Type of injury |  | | | |
| Isolated severe head injury | 23 (34%) | 14 (21%) | 9 (33%) | 0.829 |
| Multiple trauma with severe head injury | 43 (65%) | 25 (64%) | 18 (67%) |  |
| Cause of injury |  | | | |
| Road traffic incident | 37 (56%) | 21 (54%) | 16 (59%) | 0.899 |
| Incidental fall | 4 (6%) | 3 (8%) | 1 (4%) |  |
| Other non-intentional injury | 18 (27%) | 10 (26%) | 8 (30%) |  |
| Violence/Assault | 4 (6%) | 3 (8%) | 1 (4%) |  |
| Suicide attempt | 1 (2%) | 1 (3%) | 0 |  |
| Other | 2 (3%) | 1 (3%) | 1 (4%) |  |
| Pupils reactivity |  | | | |
| Bilateral unreactive | 27 (41%) | 19 (49%) | 8 (31%) | **0.019** |
| Unilateral unreactive | 8 (12%) | 7 (18%) | 1 (4%) |  |
| Bilateral reactive | 31 (47%) | 13 (33%) | 18 (65%) |  |
| Hypotension (pre-ICU admission) | 34 (52%) | 22 (58%) | 12 (44%) | 0.285 |
| ISS scale | 25 (18-41) | 33 (16-43) | 24 (20-34) | 0.516 |
| GCS-total at admission | 6 (4-8) | 5 (4-7) | 7 (6-8) | **0.012** |
| GCS-motor at admission | 4 (2-4) | 3 (2-4) | 4 (4-5) | **0.027** |
| CT characteristic |  | | | |
| Marshall CT Score | 3 (2-4) | 3 (3-4) | 3 (2-3) | **0.009** |
| Rotterdam CT Score | 3 (2-4) | 3 (2-4) | 2 (2-3) | 0.062 |
| Contusion | 36 (54%) | 22 (61%) | 14 (63%) | 0.695 |
| Edema | 51 (77%) | 33 (84%) | 18 (67%) | 0.087 |
| Subdural hematoma | 30 (45%) | 18 (46%) | 12 (44%) | 0.890 |
| Epidural hematoma | 7 (11%) | 2 (5%) | 5 (19%) | 0.083 |
| Cerebral hematoma | 13 (20%) | 5 (19%) | 8 (21%) | 0.841 |
| Diffuse axonal injury | 19 (29%) | 15 (38%) | 4 (15%) | **0.037** |
| tSAH | 37 (56%) | 25 (64%) | 12 (44%) | 0.113 |
| Other characteristics |  | | | |
| Evacuation of mass lessions | 15 (23%) | 10 (26%) | 5 (19%) | 0.497 |
| EVD or CSF drainage | 4(6%) | 3 (8%) | 1 (4%) | 0.504 |
| Decompression craniotomy | 16 (24%) | 14 (36%) | 2 (7%) | **0.007** |
| Vasopressors | 56 (84%) | 34 (90%) | 22 (82%) | 0.358 |
| MV [days] | 11 (8-23) | 14 (7-24) | 10 (8-15) | 0.123 |
| Epileptic seizures | 16 (24%) | 14 (36%) | 2 (13%) | **0.008** |
| Tracheostomy | 30 (45%) | 22 (56%) | 8 (30%) | **0.032** |
| Infectious complications | 43 (65%) | 19 (70%) | 24 (62%) | 0.459 |
| PSH probable | 9 (15%) | 9 (23%) | 0 | **0.007** |
| PSH possible | 13 (20%) | 6 (15%) | 7 (26%) | 0.289 |
| PSH unlike | 44 (65%) | 24 (62%) | 20 (74%) | 0.288 |
| Outcome |  | | | |
| ICU LOS | 21 (12-32) | 22 (9-33) | 18 (14-30) | 0.948 |
| Hospital LOS | 30 (17-48) | 31 (13-55) | 27 (20-44) | 0.866 |
| In-hospital mortality | 17 (26%) | 17 (44%) | 0 | **<0.001** |
| GCS | 14 (9-14) | 11 (8-13) | 12 (8-14) | 0.659 |

**Abbreviations**: APACHE, Acute Physiology and Chronic Health Evaluation, CSF, cerebrospinal fluid, CT, computed tomography, GCS, Glasgow Coma Scale, GOS, Glasgow Outcome Scale, ICU, Intensive Care Unit, ISS, Injury Severity Score, EVD, external ventricular drainage, LOS, length of stay, tSAH, traumatic subarachnoid haemorrhage; MV, mechanical ventilation; PSH , paroxysmal hyperactivity

**Strengthening the Reporting of Observational Studies in Epidemiology (STROBE)” statement guidelines**

|  | **Item No.** | **Recommendation** | **Page  No.** | **Relevant text from manuscript** |  |  |
| --- | --- | --- | --- | --- | --- | --- |
| **Title and abstract** | 1 | (*a*) Indicate the study’s design with a commonly used term in the title or the abstract | 1 | Heart rate variability and cerebral autoregulation in traumatic brain injury with paroxysmal sympathetic hyperactivity syndrome |  |  |
|  |  | (*b*) Provide in the abstract an informative and balanced summary of what was done and what was found | 3 | This single-centre retrospective study investigates the relationship between changes in ANS metrics, cerebral autoregulation metrics, and PSH syndrome. Arterial blood pressure (ABP) and intracranial pressure (ICP) signals were monitored for 5 days post-TBI. ANS metrics included time and frequency-domain HRV metrics. Cerebral autoregulation was assessed using the pressure reactivity index (PRx). |  |  |
| **Introduction** | | | |  |  |  |
| Background/rationale | 2 | Explain the scientific background and rationale for the investigation being reported | 4 | The outcome of TBI patients is influenced not only by the severity of brain damage but also by the extent of systemic disorders and the function of peripheral organs. Recent studies have shown that Autonomic Nervous System (ANS) disorders may significantly affect the overall outcome. The diagnosis of PSH relies on the 2014 standardized PSH-AM scoring system, which also serves as a valuable tool for assessing the severity and monitoring the effectiveness of PSH treatment. However, it is essential to acknowledge the non-specific nature of PSH-AM, posing a potential challenge in the intensive care unit (ICU) setting. |  |  |
| Objectives | 3 | State specific objectives. including any prespecified hypotheses | 6 | In this study, we investigated the relationship between ANS activity and incidence of PSH, aiming to determine the utility of autonomic metrics as significant risk factors. Moreover, we analyzed cerebral autoregulation in patients with PSH and described the relationship between ANS and cerebral autoregulation in the early period after TBI. We hypothesized that impairment of both ANS and cerebral autoregulation in the early period after TBI, in patients undergoing standard intensive care treatment, may influence poorer prognosis and morbidity. |  |  |
| **Methods** | | | |  |  |  |
| Study design | 4 | Present key elements of study design early in the paper | 6 | This study retrospectively analyzed the data of patients with moderate to severe TBI admitted to the ICU of the University Clinical Hospital in Wroclaw between 2011 and 2022. |  |  |
| Setting | 5 | Describe the setting. locations. and relevant dates. including periods of recruitment. exposure. follow-up. and data collection | 6 | This study retrospectively analyzed the data of patients with moderate to severe TBI admitted to the ICU of the University Clinical Hospital in Wroclaw between 2011 and 2022. The study was approved by the Bioethics Committee of the Wroclaw Medical University (KB-133/2023; waived the need for informed consent) and conducted according to the Strengthening the Reporting of Observational Studies in Epidemiology (STROBE) statement for observational cohort studies (Supplementary materials). |  |  |
| Participants | 6 | (*a*) *Cohort study*—Give the eligibility criteria. and the sources and methods of selection of participants. Describe methods of follow-up | 6-7 | The following inclusion criteria were used: 1) age of 18 years or older, 2) moderate or severe TBI, assessed by the Glasgow Coma Scale (GCS) as an isolated injury or predominate multi-organ injury [37], 3) implementation of the ICP sensor, 4) hemodynamically stable patient when monitoring started. Exclusion criteria were as follows: 1) previous or concurrent diagnoses that could explain symptoms of autonomic dysfunction (e.g., long-term diabetes, ischemic heart disease), 2) concurrent traumatic spinal cord injury [38], 3) lack of good-quality, high-resolution frequency signal recordings initiated within 24 hours after ICU admission. The flow chart is shown in Figure 1 |  |  |
|  |  |  | | |  |  |
| Variables | 7 | Clearly define all outcomes. exposures. predictors. potential confounders. and effect modifiers. Give diagnostic criteria. if applicable | 7-8 | The clinical short-term outcome was assessed at hospital discharge using the Glasgow Outcome Scale (GOS) [37] and defined as poor (GOS 1-3) and good (GOS 4-5). The Clinical PSH Assessment Measure (PSH-AM) was used to diagnose PSH. The PSH-AM score has two components: the clinical feature scale (CFS), to identify the intensity of cardinal features, and the diagnosis likelihood tool (DLT), to evaluate the likelihood of the presence of PSH. Combining the two sums of the total scores yields a total PSH-AM score, which assesses the probability of a PSH diagnosis. PSH-AM < 8 indicates the diagnostic probability of PSH as unlikely, 8 to 16 possible, and more than 17 as likely [45]. A detailed description of the PSH-AM scale is presented in Supplementary Table 1. Based on the clinical course, two independent investigators defined PSH as at least 3 seizure events with 2 or more simultaneous features: (1) tachycardia,(2) tachypnea, (3) hypertension, (4) fever, (5) sweating and (6) sweating dystonia, after excluding other causes (e.g., poorly controlled pain, infection, epileptic seizure). |  |  |
| Data sources/ measurement | 8* | For each variable of interest. give sources of data and details of methods of assessment (measurement). Describe comparability of assessment methods if there is more than one group | 7 | Data were digitized with an analogue–digital converter and recorded continuously at a sampling frequency of 200 Hz with the ICM+ system (Cambridge Enterprise Ltd, Cambridge, UK). Artefacts in the recordings were identified either manually or through custom-written algorithms, and further analyses were performed only on the representative parts of the signals. For patients who required decompression craniectomy, monitoring was discontinued after the surgery. Signal monitoring began within the first 24 hours of the patient’s ICU stay and continued throughout their stay. However, for this analysis, we included only the first five days of data. |  |  |
| Bias | 9 | Describe any efforts to address potential sources of bias |  | NA |  |  |
| Study size | 10 | Explain how the study size was arrived at | 6-7 | The following inclusion criteria were used: 1) age of 18 years or older, 2) moderate or severe TBI, assessed by the Glasgow Coma Scale (GCS) as an isolated injury or predominate multi-organ injury [37], 3) implementation of the ICP sensor, 4) hemodynamically stable patient when monitoring started. Exclusion criteria were as follows: 1) previous or concurrent diagnoses that could explain symptoms of autonomic dysfunction (e.g., long-term diabetes, ischemic heart disease), 2) concurrent traumatic spinal cord injury [38], 3) lack of good-quality, high-resolution frequency signal recordings initiated within 24 hours after ICU admission. The flow chart is shown in Figure 1 |  |  |
| Quantitative variables | 11 | Explain how quantitative variables were handled in the analyses. If applicable. describe which groupings were chosen and why | 10 | To demonstrate the dynamic of ANS parameters to ICP, the total group was divided into quartiles. | | |
| Statistical methods | 12 | (a) Describe all statistical methods. including those used to control for confounding | 10 | Differences in median values categorized by any dichotomized criteria were tested using the Mann–Whitney U test or Pearson’s χ2 test (or Fisher exact test) for non-numeric data. | | |
|  |  | (b) Describe any methods used to examine subgroups and interactions | 10 | The Kruskal-Wallis ANOVA was used to test the differences within those four subgroups. The relationship between ANS metrics and cerebral autoregulation was tested using Spearman’s correlation coefficient | | |
|  |  | (c) Explain how missing data were addressed |  | NA | | |
|  |  | (d) Describe any sensitivity analyses |  | NA | |  |
| Results | | | | | | |
| Participants | 13* | (a) Report numbers of individuals at each stage of study—eg numbers potentially eligible. examined for eligibility. confirmed eligible. included in the study. completing follow-up. and analyzed | 11 | Between 2011 and 2022, 528 patients with TBI were admitted to the ICU of the Wroclaw University Hospital and evaluated for inclusion in the study. Of these, 66 subjects (27% female) were included. The flow chart is presented in Figure 1, and clinical characteristics are detailed in Table 1. The median age of the cohort was 33±24 years. | | |
|  |  | (b) Give reasons for non-participation at each stage | 11 | The flow chart is presented in Figure 1, and clinical characteristics are detailed in Table 1. | | |
|  |  | (c) Consider use of a flow diagram | 11 | The flow chart is presented in Figure 1, and clinical characteristics are detailed in Table 1. | | |
| Descriptive data | 14* | (a) Give characteristics of study participants (eg demographic. clinical. social) and information on exposures and potential confounders |  | Table 1 | | |
|  |  | (b) Indicate number of participants with missing data for each variable of interest |  | Table 1 | | |
|  |  | (c) Cohort study—Summaries follow-up time (eg. average and total amount) |  | Figure 1 | | |
| Outcome data | 15* | Cohort study—Report numbers of outcome events or summary measures over time | 11 | 17 subjects (26%) died in the hospital, where irreversible brain damage was the main cause of death. The comparison of clinical characteristics between patients with isolated head trauma (n=23, 35%) and those with polytrauma (n=43, 65%) is presented in Table 1. Impaired cerebral autoregulation was found in 48 patients (73%). PSH syndrome was classified as likely in 9 (14%) subjects and possible in 13 cases (20%). | | |
|  |  | Case-control study—Report numbers in each exposure category. or summary measures of exposure |  |  | | |
|  |  | Cross-sectional study—Report numbers of outcome events or summary measures |  |  | | |
| Main results | 16 | (a) Give unadjusted estimates and. if applicable. confounder-adjusted estimates and their precision (eg. 95% confidence interval). Make clear which confounders were adjusted for and why they were included | 13 | In the multiple logistic regression model for PSH, HRV LF and HR were adjusted by standard clinical factors (age, sex, GCS), and risk factors for PSH syndrome (fever, diffuse axonal injury, polytrauma). The final model (CHI^2^ = 8.59, p=0.014) is detailed in Table 3. It included diffuse axonal trauma (OR =10.82 (95% CI 1.70-68.98, p=0.012) and HR (OR =0.91 (95% CI 0.84-0.89, p=0.021). The model demonstrated moderate prediction ability (AUC = 0.84), as illustrated in Figure 4B.  In the multiple logistic regression model for mortality, HRV LF/HF, SDNN, and RMSSD were adjusted by standard clinical factors (age, sex, GCS). The final model (CHI^2^ = 10.72, p=0.004) is presented in Table 3. It included HRV LF/HF (OR = 0.23 (95% CI 0.07-0.75, p=0.014) and RMSSD (OR = 1.05 (95% CI 1.01-1.08, p=0.006). This model demonstrated moderate prediction ability (AUC = 0.80), see Figure 4B. | | |
|  |  | (b) Report category boundaries when continuous variables were categorized |  |  | | |
|  |  | (c) If relevant. consider translating estimates of relative risk into absolute risk for a meaningful time period |  |  | | |
| Other analyses | 17 | Report other analyses done—eg analyses of subgroups and interactions. and sensitivity analyses |  | Supplementary Data | | |
| Discussion | | | | | | |
| Key results | 18 | Summaries key results with reference to study objectives | 14 | Our study demonstrated that ANS disorders in the early stages of TBI are associated with the development of PSH syndrome. To the best of our knowledge, this study is among the first to evaluate the utility of HRV assessment and cerebral autoregulation based on continuous monitoring in acute TBI for the early prediction of PSH syndrome. We found that a high LF, reflecting modulation of cardiac autonomic outflows by baroreflexes [54], and a low HR were moderate, independent early predictors of PSH development. In the adjusted model of PSH, we found that HR and diffuse axonal injury emerged as significant factors influencing the development of PSH. Notably, standard clinical risk factors, such as fever, age, and polytrauma were not important predictors. Additionally, we found that a decreased LF/HF ratio, along with higher RMSSD and SDNN contributed to an increased risk of death, underscoring the importance of ANS monitoring in TBI patients. Although we noted impairment in cerebral autoregulation in comparable ratio both in patients with PSH as well as in patients with possible/unlikely PSH, the impact of those regulatory mechanism requires further studies. | | |
| Limitations | 19 | Discuss limitations of the study. taking into account sources of potential bias or imprecision. Discuss both direction and magnitude of any potential bias | 17 | This study has limitations. TBI may disrupt the autonomic system’s response to shock, particularly in cases of polytrauma [85,86]. Although our study group consisted of hemodynamically stable patients, this aspect should be acknowledged. In our analysis, we used data collected from patients who were hospitalised several years ago. Nevertheless, patients’ management followed standardised protocols that were close to the recently published treatment guidelines [40,41]. Our cohort of patients was homogeneous in terms of mechanical ventilation and medications. The majority of the group was under the influence of vasopressors with propofol being the main sedative. It should be noted that recent studies have shown that the supply of sedatives, analgesics and vasopressors has no clinically significant effect on cerebral vascular reactivity [87,88]. The limited number of subjects in the subgroup analysis may introduce bias and reduce the statistical power of the test. Therefore, these results need to be confirmed in a large multicentre study. | | |
| Interpretation | 20 | Give a cautious overall interpretation of results considering objectives. limitations. multiplicity of analyses. results from similar studies. and other relevant evidence | 17 | In our study, we did not observe any significant changes in the daily averages of ANS parameters. However, analysing temporary patterns and daily variation in ANS seems to be a promising approach [51]. Previous studies have shown that even short episodes of hypotension or suboptimal CPP level can significantly impact neurological outcome, thus offering valuable prognostic information [83]. | | |
| Generalizability | 21 | Discuss the generalizability (external validity) of the study results | 16 | Impairment in cerebral autoregulation has been reported as an independent predictor of mortality in acute brain injury [51,68–71], and changes in cerebral autoregulation, along with alterations in serum biomarkers levels, may predict in cerebral ischemia [72]. However, the interplay between ANS disorders and disruptions in cerebral autoregulation is a subject of debate [70]. Previous studies on TBI patients have either focused on abnormal autoregulation of cerebral circulation [70,73–75] or analyzed HRV metrics during the acute phase of TBI in relation to treatment outcomes [9,16,17,76,77]. | | |
| Other information | | | | | | |
| Funding | 22 | Give the source of funding and the role of the funders for the present study and. if applicable. for the original study on which the present article is based. | 2 | This work was supported by the National Science Centre, Poland (grant no UMO-2022/47/D/ST7/00229) and the Research Committee of the Medical University of Wroclaw Research no: SUBK.A170.23.048 | | |

*Give information separately for cases and controls in case-control studies and. if applicable. for exposed and unexposed groups in cohort and cross-sectional studies.

Note: An Explanation and Elaboration article discusses each checklist item and gives methodological background and published examples of transparent reporting. The STROBE checklist is best used in conjunction with this article (freely available on the Web sites of PLoS Medicine at http://www.plosmedicine.org/. Annals of Internal Medicine at http://www.annals.org/. and Epidemiology at http://www.epidem.com/). Information on the STROBE Initiative is available at www.strobe-statement.org
